# Parkinsonism and chronic manganese exposure: Pilot study with clinical, environmental and experimental evidence

**DOI:** 10.1016/j.prdoa.2020.100057

**Published:** 2020-05-06

**Authors:** Najib Kissani, Yahya Naji, Yassine Mebrouk, Mohamed Chraa, Abderrazzak Ghanima

**Affiliations:** a Clinical and Experimental Neuroscience Research Laboratory, Faculty of Medicine, Cadi Ayyad University, Marrakech, Morocco; b Neurology Department, University Hospital Mohammed VI, Oujda, Morocco; c Laboratory of Bio-Organic and Macromolecular Chemistry, Department of Biology, Faculty of Sciences and Techniques, Marrakech, Morocco; d Neurology department, University Teaching Hospital Mohammed VI, Marrakesh, Morocco

**Keywords:** Parkinsonism, Manganese, Chronic exposure, Toxicological samples

## Abstract

Parkinsonism related to chronic Manganese exposure is notably due to focal lesions of the basal ganglia. Our study focused on epidemiological, clinical, toxicological and experimental aspects of Manganese-induced Parkinsonism in south of Morocco. It is a prospective study concerning the workers and the residents bordering on the 2 mines in the south of Morocco. The results of the study concerned 120 cases divided into 4 groups of patients: G1: 30 cases exposed to different incriminated toxic products, which present Parkinsonian signs, G2: 30 cases healthy and exposed, G3: 30 cases affected with Idiopathic Parkinson's disease, and G4: 30 cases healthy and unexposed (controls).

The results from the first mine show that 5.7% of the sample developed Manganese-Induced Parkinsonism and this percentage is slightly higher (4.5%) than the second mine site. Chemical and biological analysis revealed high levels of Manganese. The majority of patients did not improve the clinical signs under L-dopa treatment. The authors underline the gravity of Manganese-induced Parkinsonism and propose a listing of the various exposures as well as a cartography of the regions of risk in Morocco.

## Highlights

• The objective of this study was to update the data about Manganese-Induced Parkinsonism in the South of Morocco.
• Multidisciplinary study putting together clinicians, biologists, biochemists, and pathologists to map accurately areas of exposure to heavy metals.
• Specific considerations should be taken concerning miners by improving work conditions and obliging the use of protective equipment.
• General approach on primary prevention for the population living near the mines in the way to decrease the number of cases.

## 1 Introduction

Parkinsonism is a term that clinically describes patients with tremor, bradykinesia, rigidity, and gait balance problems, which are symptoms of dysfunction of the basal ganglia [1]. Manganese is an essential metal for humans and daily requirements are commonly met by an adequate diet. However it turn to be a basal ganglia toxin upon excessive exposure, causing damage to the striatal “direct pathway” (the caudate/putamen, internal globus pallidus, and substantia nigra pars reticulata) with a marked inhibition of striatal dopamine release [2].

Manganese-induced Parkinsonism differs from the idiopathic Parkinson's disease (iPD), which typically is characterized by bradykinesia and rigidity that responds well to the L_dopa treatment and a loss of postural stability occurring only in later stages of the disease [2]. Several studies are underway to elucidate the etiology of this disease, and so far no specific cause has been identified. Although a combination of environmental and genetic factors are thought to contribute to the expression of Parkinsonian phenotype [3,4]. Nowadays, few epidemiological and toxicological studies have been conducted on the subject in Africa in general and Morocco in particular [5,6]. The first studies in Morocco, carried out in 1955 by Rodier [7], had demonstrated manganese poisoning with clinical characteristics dominated by extra-pyramidal symptoms, including dystonic postures in miners in southern Morocco.

Many articles treat the relation between Parkinsonism and manganese exposure especially welding, ferroalloy and mining [8,9]. But few perform a toxicological study on both biological and environmental samples. They even show that exposure to manganese dust and fumes might produce toxin-related Parkinsonism. However a debate exists on whether chronic exposures in welding, ferroalloy and other industrial processes could lead to significant neurobehavioral changes such as Parkinsonism [10].

This study based on an analytical and comparative methodology between 4 groups of 120 cases, we will address the following issues: what's the prevalence of Parkinsonism among mines residents, who are the most affected, what are the most incriminated metals involved in parkinsonism, is there a real contamination in both biological samples (blood, urine) and samples taken from exposure sites (soil, water), what are the nature of the nervous system dysfunctions caused by chronic Manganese exposure.

Our study will show the persistence of Manganese-induced parkinsonism cases in Southern Morocco and expose the environmental impact of Moroccan manganese mines and sort out practical conclusions and recommendations to take care and protect the high-risk populations and the environment.

## 2 Methods

### 2.1 The study design

Our prospective study covered the period between February 2005 and October 2009. It involves 3 steps and took place at various selected sites based on epidemiological and clinical data as well as mine mapping present in the south of Morocco. It consisted of:

– Epidemiological and clinical studies of all participants with a collection of informations about their family and socio-professional environment.
– Biochemical and toxicological studies of the different samples from participants (blood, urine, and hair) and environment (water, soil, plants)

### 2.2 Participants

**Fig. 1:**
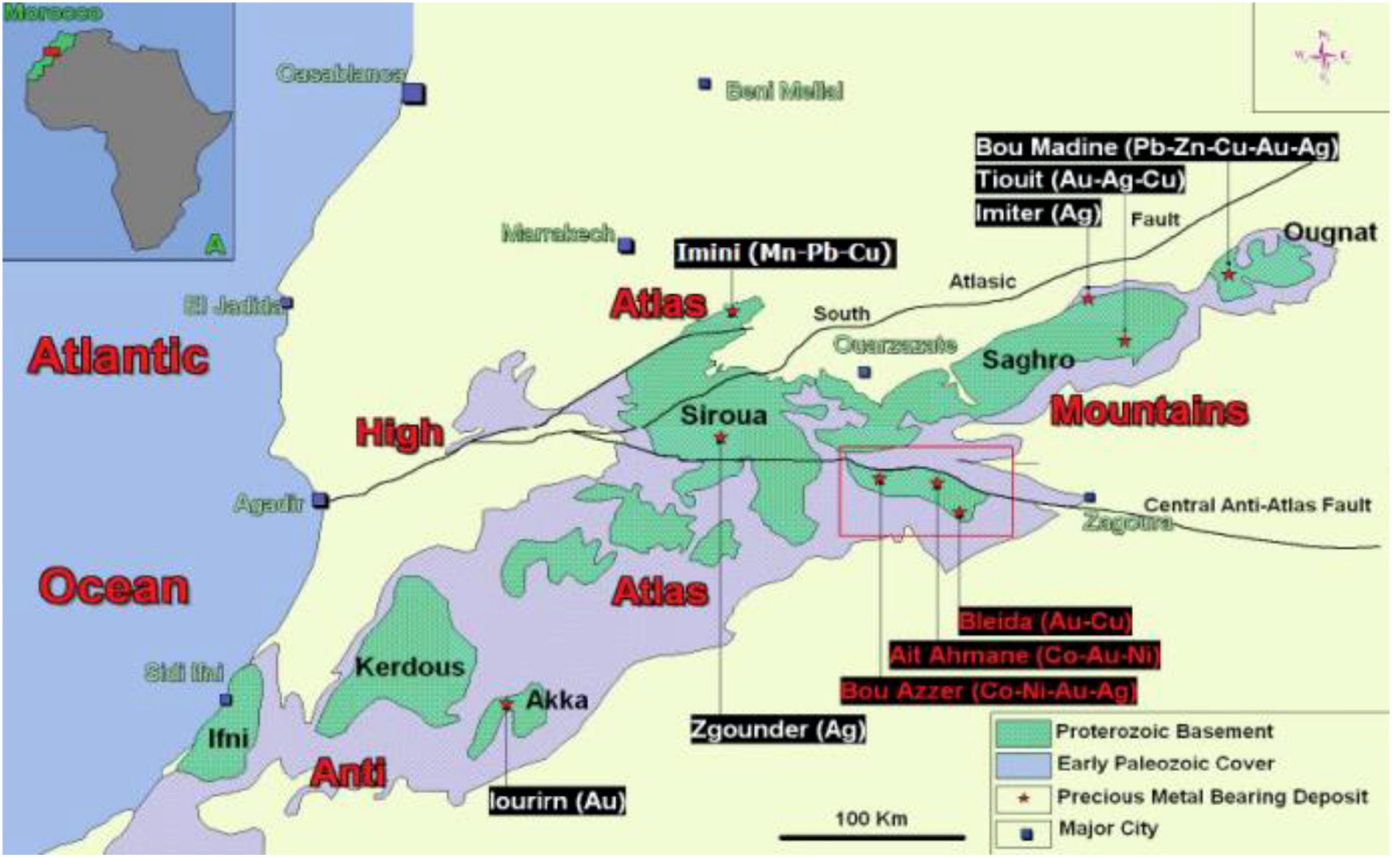
Distribution of Metalliferous sites/mines particularly located in southern Morocco [11].
▪ Group 2 (G2): 30 participants exposed to metals under investigation, living in the visited sites and having different occupations (miners, welders, bordering residents), had a normal clinical examination.
▪ Group 3 (G3): 30 participants with Idiopathic Parkinson's disease are recruited from the Neurology consultation at the University Hospital Mohammed VI, Marrakech, Morocco. And they are not exposed to any of the metals under investigation.
▪ Group 4 (G4): 30 cases healthy and unexposed, the team of the Neurology department with neuroscientists from Science & techniques faculty of Marrakech served as controls for the blood test).

120 participants were selected for the study and divided into four groups of 30 persons each:

▪ Group 1 (G1): 30 participants exposed to metals under investigation, living in the visited sites and having different occupations (miners, welders, bordering residents), the 30 ones showed signs of Parkinsonism after being examined by the neurology team of the University Hospital Mohammed VI, Marrakech, Morocco. Theses visited sites are the mine of Imini (30 km North of Ouarzazate) and mine of Kettara (34 km North-West of Marrakech) (Fig. 1).

All participants were informed about the aim and procedures of the study; they gave their informed consent and participated voluntarily. Clinical data were collected using an exploitation sheet specifying socio-demographic information, the way of life, the exposure factors, functional signs, and results of the neurological assessment.

To appreciate the degree of severity and evolution we used the UPDRS scorings and the Hoehn-Yahr stage, we also practiced the L-dopa test on participants from the first and third group.

Data collections were done during several visits of concerned regions directed by the team of neurology department in the University Hospital Mohammed VI, Marrakech, Morocco with neuroscientists from Science & techniques faculty of Marrakech. Those visits were supported by the ministry of health delegation in coordination with local authorities.

### 2.3 Biological samples

Blood test of the 120 participants was done by using a plastic syringe fitted with a stainless- steel needle from antecubital vein. Additionally, urine was obtained from each participant, simultaneously to the blood extractions. The collected samples underwent an analysis by atomic absorption spectroscopy to identify and quantify the metals under investigation (Manganese, Copper, Zinc, and Lead).

### 2.4 Environmental samples

Water and soil samples were simultaneously taken from the 3 visited sites. The stream waters samples were centrifuged and soil were air-dried, homogenized, and sieved through a 2-mm plastic screen before chemical analyses. Groundwater was sampled from selected wells located in the area of the 3 mining sites. All the wells have been selected according to their proximity to the mine and their use (drinking or irrigation). The groundwater samples were passed through cellulose-nitrate membrane filters before chemical analyses. Metal analysis was done by a Flame Atomic Absorption Spectrometry (A.A.S.) according to the methods described in the literature (French Association of Normalization (AFNOR) 1983).

### 2.5 Statistical analysis

Statistical analyses were carried out with the software Microsoft Excel for Windows. The statistical analyses consisted at first of describing the socio-demographic variables, clinical and patient outcomes to determine the prevalence of Manganese-induced parkinsonism. In the second step, comparative analyses were carried out between the four groups.

## 3 Results

The first prospected mine was the Imini mine located in the area of the Anti-Atlas, near the south of the Atlas, on the road from Marrakech to Ouarzazate. The Imini site is the most important Moroccan deposit of manganese with total production cumulated since it opened in 1938 of nearly 7 million tons of manganese Pyrolusite (a mineral containing mainly manganese dioxide Mn0_2_) represent 72% of merchant ore. The mineralogical association includes impurities, Lead, Copper, Iron, Phosphorus, Cobalt, Nickel, and Arsenic [11].

The second prospected site was the Kettara mine located in the heart of Kettara village located 34 Km at the North West of Marrakech on the road Marrakech-Safi.

Extraction from this mine started in 1930; since 1982, the waste from this mine has been deposited over a very large area near the village. The main product is pyrrhotite, inorganic iron sulfide with the formula Fe_(1-x)_ S. The mineralogical association includes iron oxide, Cooper and Zinc [11].

Miners, workers, and bordering residents of the 2 mines were examined by the neurology team of the University Hospital of Marrakech in collaboration with local authorities. The socio-demographic characteristics and the prevalence of all Parkinsonism cases of the 3 groups are summarized in Table 1.

**Table 1:** The socio-demographic characteristics with the prevalence of different Parkinsonism form.

Item	G1^a^		G2^a^	G3^a^	G4^a^
Average age (years)	39		37	59	34
Gender M/F	29/1		28/2	22/8	19/11
	1st mine	2nd mine	1st mine+2nd mine		
Sample/general population (n°/N)	192/5711	46/1527	238/7238	–	–
Number of Parkinsonism cases (prevalence)	17 (8.8%)	6 (7.5%)	–	30	–
Manganese-induced Parkinsonism	11 (5.7%)	2 (4.5%)	–	0	–
Other atypical Parkinsonism form	6 (3.1%)	4 (8.7%)	–	0	–
Idiopathic Parkinsonism	0	0	–	30	–

a Group 1 (G1): 30 participants exposed to different incriminated toxic products, living in the visited sites and shows signs of Parkinsonism after been examined, Group 2 (G2): 30 participants exposed to different incriminated toxic products, living in the 2 visited sites and don't show any Parkinsonism signs (Neurological assessment was normal), Group 3 (G3): 30 participants presenting Idiopathic Parkinson's disease are recruited from the Neurology consultation at the University Hospital of Marrakech. Group 4 (G4): 30 healthy and unexposed participants, they were the team of the Neurology department with Neuroscientists from Science & techniques faculty of Marrakech (witnesses for blood samples).

**Fig. 2:**
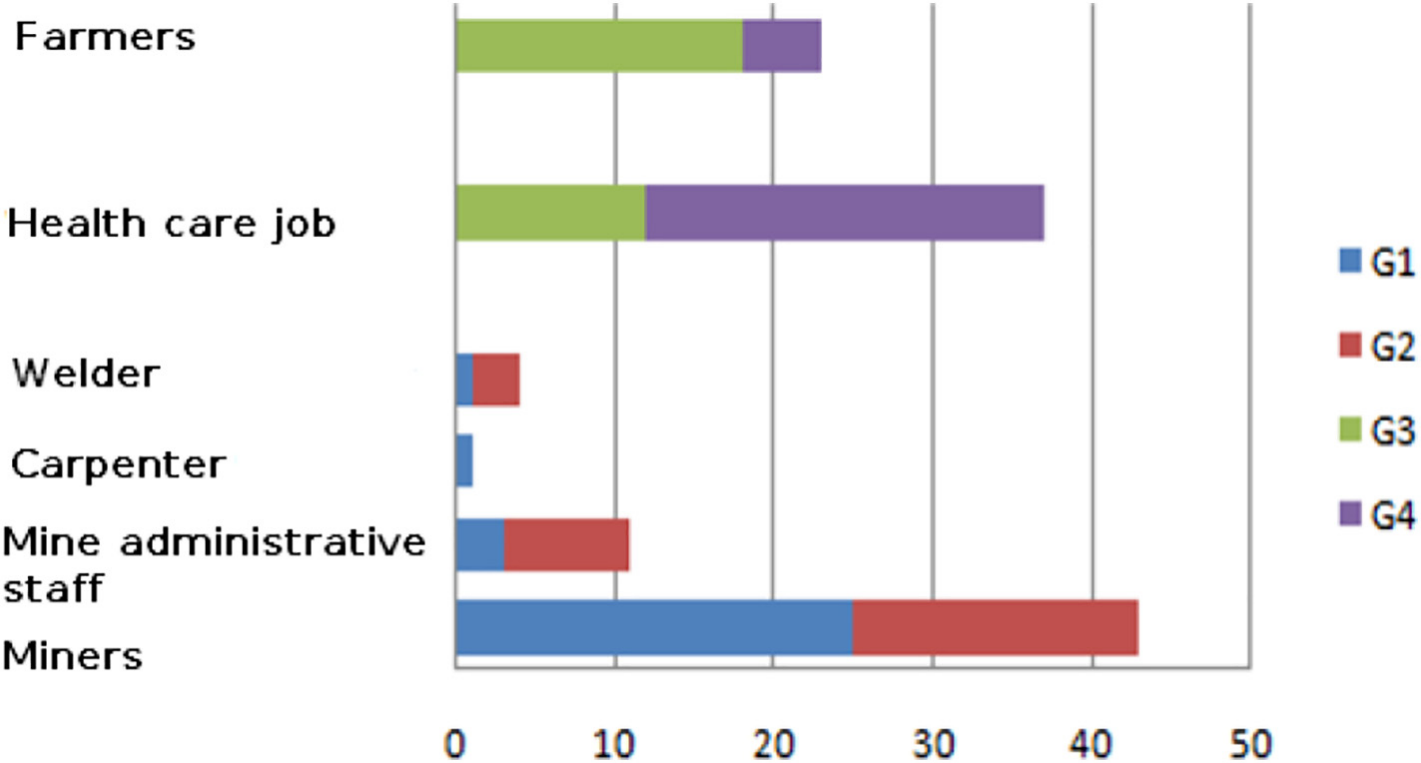
Distribution of the four groups according to occupation.

**Fig. 3:**
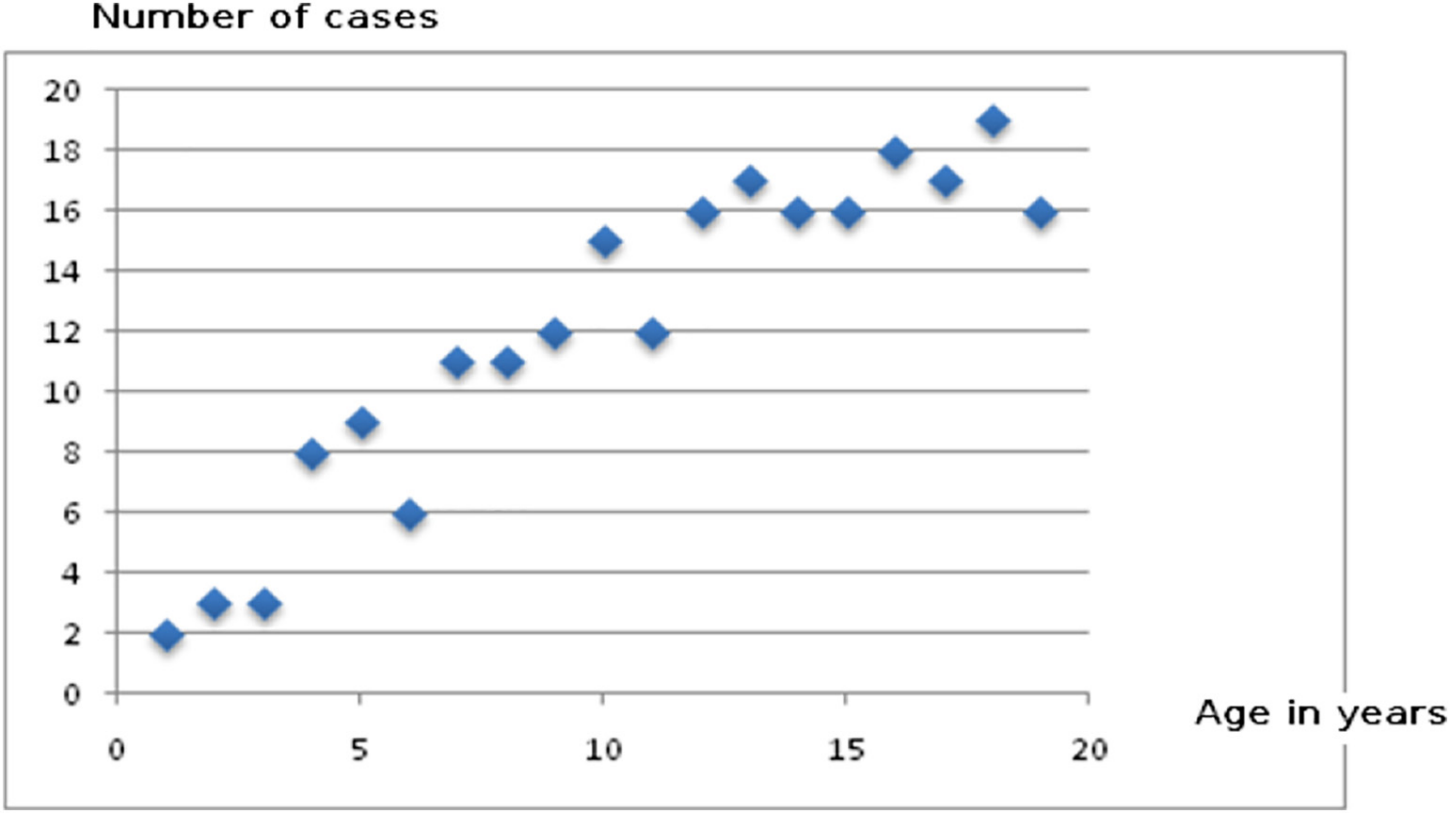
Number of subjects with parkinsonism in relation to the number of years worked in the mine.

The population, suffering from more cases of Manganese-Induced Parkinsonism, was directly linked to the type of occupation. The miners were mainly the most exposed to the different metals under investigation (Mn0_2_, FeS, Cu, Zn, Pb) and displayed more cases of Manganese-Induced Parkinsonism with a percentage of 65%, compared to other occupations like mine administrative staff with only 26% of Manganese-Induced Parkinsonism cases (Fig. 2). The prevalence of parkinsonism cases increases in relation to the number of years worked in the mines (Fig. 3). Mostly akinetic forms distinguish Manganese-Induced Parkinsonism in our study, while akinetic and tremor forms characterize the third group (Table 2).

**Table 2:** Distribution of subjects according to Parkinsonism form with the percentage of each form in G1 and G3.

Item	G1	G3
Akinetic form	19 (31%)	5 (8%)
Tremor form	5 (8%)	4 (6%)
Akinetic and tremor form	6 (10%)	21 (35%)

**Fig. 4:**
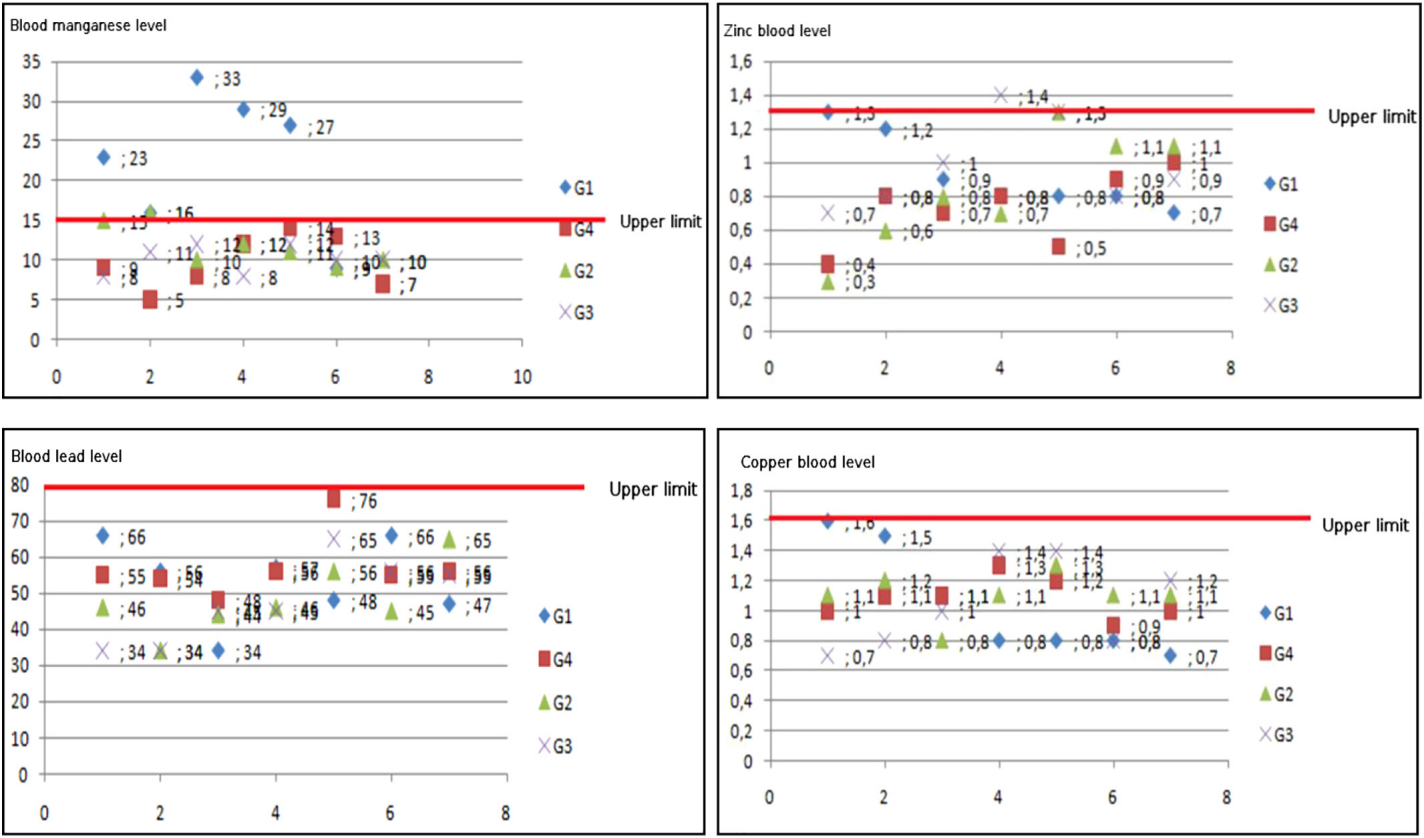
Blood screening of Manganese (Upper limit is >15 μg/L), Zinc (Upper limit is >1.30 μg/mL), Cooper (Upper limit is >1.6 μmol/L), Lead (Upper limit is >80 μg/dL) (*n* = 30).

A toxicological study was conducted in all patients and focused on the determination of heavy metals, particularly Manganese, Copper, Zinc, and Lead, in blood, and urine. Soil and water samples were analyzed for the same metals. Blood screening showed high levels of Manganese in the first group (G1) and some cases of the second group (G2), other heavy metals (Cu, Zn, Pb) are found at normal rates (Fig. 4). The biological samples analysis (soil/water) show that stream waters heavily enriched with Manganese contribute to the contamination of the soils especially the closest to the tailings deposits of heavy metals (Mn concentration of soils was between 507 and 3462 mg/kg). However, most of the groundwater studied had relatively low Manganese concentrations.

## 4 Discussion

The prevalence of Manganese-induced Parkinsonism is unknown, only small number of cases have been reported and there has been no epidemiological assessment of various workplaces or occupations [1]. However, In 1955, Rodier had found in southern Moroccan miners, a manganic intoxication with clinical symptoms dominated by the extrapyramidal symptomatology [7,12,13].

Another study show cases of Manganese-Induced Parkinsonism due to Ephedrone Abuse which is a drug mixture containing methcathinone and manganese [2,14]. Recent study show that contaminated air or water lead to Manganese intoxication, which it has been associated to a higher prevalence of Parkinsonian disturbances [15] and to cognitive troubles like poorer memory and attention, with hyperactive behavior in school-aged children [16].

Our work is the first study of its kind carried out in North Africa. The results show a higher prevalence compared to other regions of Morocco (Marrakech/Casablanca), 5,7% of the population presents Manganese-Induced Parkinsonism in the Imini mine (extracting mainly Pyrolusite Mn0_2_) and 4.5% at Kettara mine (extracting mainly Pyrrhotite Fe_(1-*x*)_S).

The main metals involved in the genesis of toxic parkinsonism is Manganese, based on the highest prevalence of cases at the first mine which represent the important Moroccan deposit of Manganese compared to the second mine (no Manganese extracting). Add to that the Blood screening of Manganese shows a high level upper than 15 μg/L at the two first groups (G1 and G2), moreover the different biochemical studies of the environment (soil, well water) confirm a widespread pollution which increases the risk of Manganese intoxication. Our findings about Manganese-induced Parkinsonism cases concord perfectly with many similar publications [1,2,8,10,17], first of all the young age of the patients (average age is 39), the predominance of akinetic form, the weak response to dopa therapy. The latency between the beginning of the exposure and the appearance of the first symptoms of Manganism is very variable, but most cases had a duration of exposure >5 years. MRI scan was performed for some cases and it show's in T1 high signal intensity with a normal T2 signal, Flair, diffusion and did not take the contrast on the Pallidums, sometimes extend to Putamen, Caudate Nucleus and the Locus Niger. We did not found significance correlation between blood levels of Manganese and the intensity of the T1 signal in Pallidums on MRI scan [1,2,17].

Manganese is the fifth most abundant metal found in the environment, >8 millions tons are extracted annually, 90% of it goes to the manufacturing of alloys, steel, iron, glass ceramics and fertilizers [18,19]. The main industrial activities causing an important exposure of Manganese to workers are the Manganese extraction and the metallurgical industry [9,10,15]. However occupational Mn-induced parkinsonism may occur after prolonged inhalation of Manganese fumes and dusts [15,20]. Manganese is transported into cells by a number of plasmatic transporters [20]. When homeostatic regulation capacity based mostly on ferroportin and SLC30A10 is exceeded [21]. The elevated intracellular levels of Manganese induce progressive neuronal degeneration of the globus pallidus mediated by disruption of the mitochondria initiating both apoptosis and cell death via formation of highly reactive oxygen species [1,17,21]. Manganese induce alterations of motor coordination, emotional and cognitive dysfunction because it affects differents neurotransmitter systems such as the Dopaminergic system [22], GABAergic system [23], Glutamatergic systems [24], and the cholinergic system [20].

In our study some cases from the second group (G2) present a high level of Manganese in their blood without any signs of Parkinsonism, while other cases from the first group (G1) shows signs of Manganese-induced Parkinsonism with a short duration of exposure (<1 year), So authors have shown that only environmental exposure isn't enough and there is other factors like genetic susceptibility or genetic initiation of neurotoxic aggression by endogenous Manganese [2]. Indeed recent genetic studies have shown that homozygous mutations in SLC30A10 who play a fundamental role in protecting against Manganese toxicity [21]. Which lead to the onset of a familial Manganese-induced parkinsonism because mutants proteins stay trapped in the endoplasmic reticulum and fail to export Manganese out of the cell [2,21]. Until now mutations in SLC30A10 are the only genetic factor known to be associated with a familial Manganese-induced Parkinsonism, and these results highlight the importance of SLC30A10 and efflux in regulating Manganese levels at the cellular and organismal level [21,25].

## 5 Conclusion

This first multidisciplinary study in Morocco and North Africa brings clinicians, biologists, and biochemists together in order to demonstrate the existence of Manganese-induced Parkinsonism in southern Morocco. The authors highlight the frequency and severity of toxic exposures to heavy metals, particularly Manganese.

The risk of Manganese exposure is not limited to miners or welders, The contamination of the environment (water or food) with high concentration of Manganese represents a source of contamination for the general population.

Manganese-induced Parkinsonism result from a combination of chronic exposure (occupational or environmental) and genetic susceptibility (SLC30A10 mutation). However, More researches are necessary to better elucidate the pathogenesis of Manganese neurotoxicity. The possible impact of manganism across Morocco must be assessed along with the prevention of environmental and occupation exposure to metals, especially Manganese.

## CRediT authorship contribution statement

**Najib Kissani:** Conceptualization, Methodology, Software, Data curation, Writing - original draft, Visualization, Investigation, Supervision, Validation, Writing - review & editing. **Yahya Naji:** Data curation, Writing - original draft, Writing - review & editing. **Yassine Mebrouk:** Data curation, Writing - original draft, Visualization, Investigation. **Mohamed Chraa:** Data curation, Writing - original draft, Software, Validation, Writing - review & editing. **Abderrazzak Ghanima:**Visualization, Investigation, Supervision, Software, Validation, Writing - review & editing. **Jacques Reis:** Supervision, Software, Validation, Writing - review & editing.

## Declaration of competing interest

This research did not receive any specific grant from funding agencies in the public, commercial, or not-for-profit sectors.
